# 1536. Acceptability of an HIV Pre-Exposure Prophylaxis (PrEP) Shared Decision-Making Tool for Diverse Populations and Healthcare Providers

**DOI:** 10.1093/ofid/ofad500.1371

**Published:** 2023-11-27

**Authors:** Wendy W Davis, Andrea Mantsios, Aimee A Metzner, Ali Talan, Jennafer Kwait, Humberto Gonzalez Rodriguez, Tahilin Sanchez Karver, Suyanna L Inhales Barker, Angela Suarez, Ricardo F Fernandez, Carlos Rodriguez-Diaz, Tamara Taggart, Alan Oglesby, Cindy Garris, David A Wohl, Clare Barrington, Deanna Kerrigan

**Affiliations:** George Washington University Milken Institute School of Public Health, Washington, District of Columbia; Public Health Innovation and Action, New York, New York; ViiV Healthcare, Durham, North Carolina; Whitman-Walker Institute, Washington, District of Columbia; Whitman-Walker Institute, Washington, District of Columbia; University of North Carolina at Chapel Hill, Durham, North Carolina; Johns Hopkins Bloomberg School of Public Health, Baltimore, Maryland; La Clinica del Pueblo, Washington, DC, District of Columbia; La Clinica del Pueblo, Washington, DC, District of Columbia; La Clinica del Pueblo, Washington, DC, District of Columbia; George Washington University-Milken Institute School of Public Health, Washington, District of Columbia; George Washington University, Washington, District of Columbia; ViiV Healthcare, Durham, North Carolina; ViiV Healthcare, Durham, North Carolina; University of North Carolina at Chapel Hill School of Medicine, Chapel Hill, North Carolina; UNC Gillings School of Global Public Health, Chapel Hill, North Carolina; George Washington University, Washington, District of Columbia

## Abstract

**Background:**

Shared decision-making tools (SDT) support conversations between healthcare providers (HCPs) and patients around evidence-based care options. With the FDA approval of long-acting injectable (LA) pre-exposure prophylaxis (PrEP) creating the choice of daily oral or LA PrEP for HIV prevention, formative research was conducted to inform the development and assessment of a prototype PrEP SDT.

**Methods:**

People who may benefit from PrEP (PWBP) (n=41) and PrEP HCPs (n=20) participated in in-depth interviews (IDIs) in Spanish or English at two sites in Washington, D.C. to explore knowledge and perceptions of daily oral and LA PrEP through 2021 and 2022. Based on input from IDIs, including on desired SDT content, a prototype PrEP SDT was developed and piloted in 37 PWBP-HCP mock encounters, including ten in Spanish, at sites in D.C. and North Carolina through 2022 and 2023. Field notes from post-encounter exit interviews were analyzed to identify content and format refinement as well as implementation implications. Matrices were used to synthesize and compare findings across populations and sites.

**Results:**

A diverse sample of PWBP and HCPs participated in IDIs (**Table 1**) and exit interviews (**Table 2**). Participants found the language, visuals, content and flow of the prototype SDT to be broadly acceptable with minor suggestions for additional content. PWBP felt the SDT addressed knowledge deficits about PrEP and that the format aided PrEP decision-making. HCPs liked that the SDT systematized clinic visits and was appropriate for never and ever PrEP users. The SDT was seen to normalize PrEP conversations and reduce stigma around PrEP use by facilitating non-judgmental and more interactive dialogue. Themes related to implementation included: the potential benefit of reviewing the SDT prior to a clinical visit and provider orientation on SDT use; the value of access to additional details on side effects and research on oral and LA PrEP; and the importance of PWBP-HCP dynamics.

**Figure:**
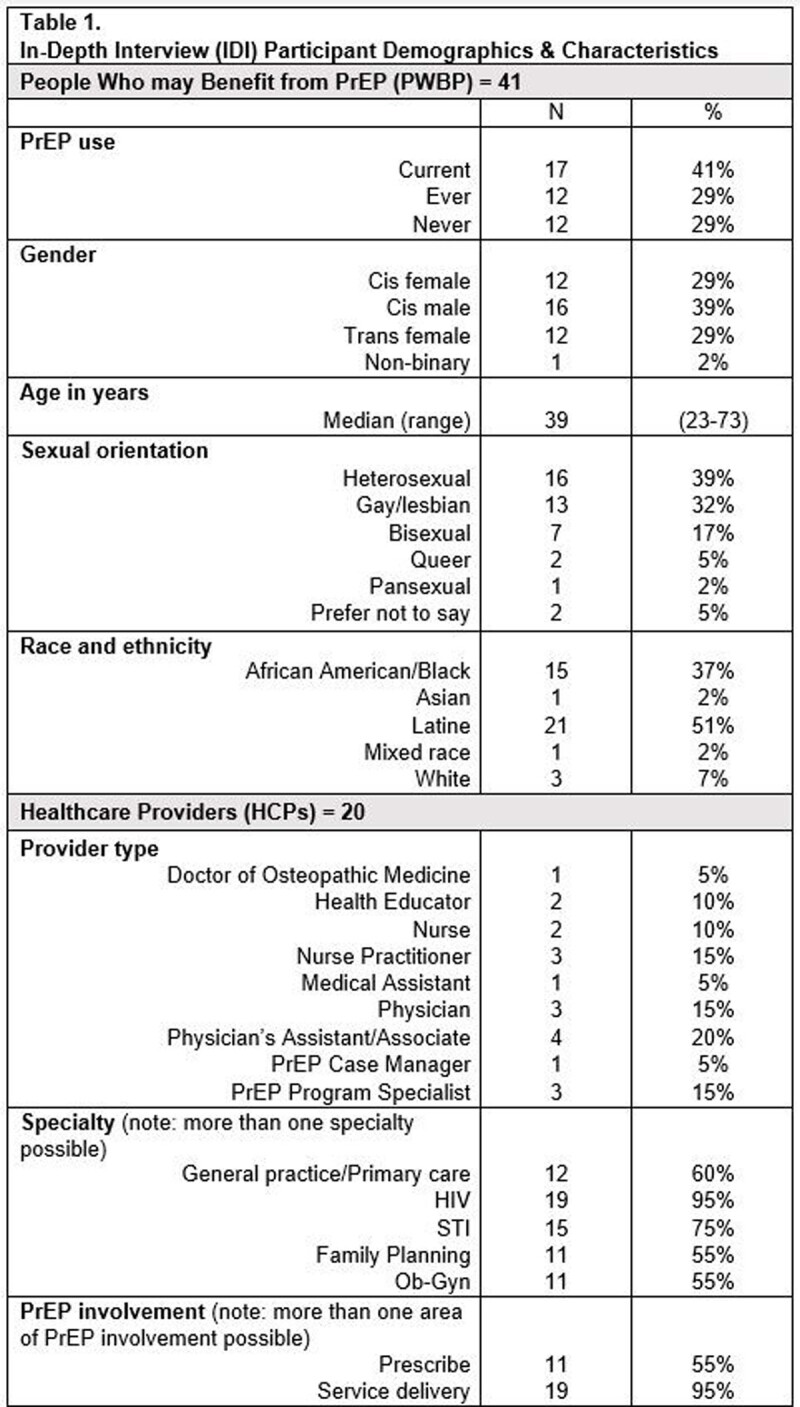

**Figure:**
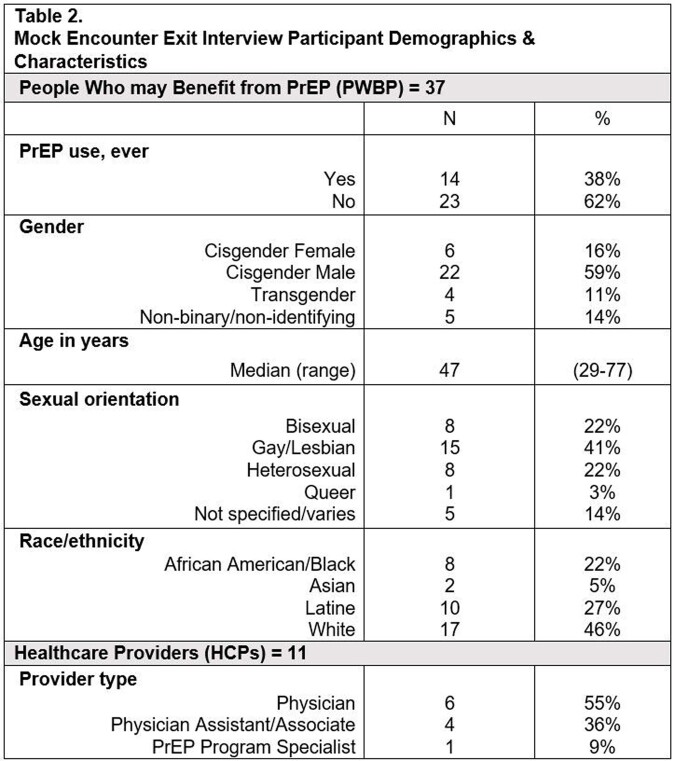

**Conclusion:**

The prototype PrEP SDT supported PrEP knowledge and choice for both ever and never PWBP while reducing stigma around PrEP use across settings and populations. It provided HCPs with content and a format that ensured comprehensive, acceptable delivery of PrEP choice information.

**Disclosures:**

**Aimee A. Metzner, PharmD, AAHIVP**, ViiV Healthcare: Full-time employee (salary/benefits/etc.)|ViiV Healthcare: Stocks/Bonds **Alan Oglesby, MPH**, GlaxoSmithKline: Employment|GlaxoSmithKline: Stocks/Bonds **Cindy Garris, MS**, GSK: Stocks/Bonds|ViiV Healthcare: Employee **David A. Wohl, M.D.**, Gilead: Advisor/Consultant|Gilead: Grant/Research Support|Janssen: Advisor/Consultant|Theratech: Advisor/Consultant|ViiV: Advisor/Consultant

